# In-Vitro Growth Inhibition of Bacterial Pathogens by Probiotics and a Synbiotic: Product Composition Matters

**DOI:** 10.3390/ijerph17093332

**Published:** 2020-05-11

**Authors:** Jacek Piatek, Hanna Krauss, Arleta Ciechelska-Rybarczyk, Malgorzata Bernatek, Paulina Wojtyla-Buciora, Henning Sommermeyer

**Affiliations:** 1 Department of Medicine, The President Stanisław Wojciechowski State University of Applied Sciences in Kalisz, Nowy Šwiat 4, 62-800 Kalisz, Poland; hjk12@poczta.fm (H.K.); paulinawojtyla@gmail.com (P.W.-B.); h.sommermeyer@pwsz-kalisz.edu.pl (H.S.); 2 District Sanitary-Epidemiological Station in Jarocin, Waska 2, 63-200 Jarocin, Poland; mikrobiologia@psse-jarocin.pl; 3 State Hospital Jarocin, Szpitalna 1, 63-200 Jarocin, Poland; drpiatek@o2.pl

**Keywords:** gut microbiome, antimicrobial activity, pathogen overgrowth

## Abstract

A variety of activities potentially contribute to the beneficial effects of probiotic bacteria observed in humans. Among these is a direct inhibition of the growth of pathogenic bacteria in the gut. The present study characterizes head-to-head the in-vitro pathogen growth inhibition of clinically relevant infectious bacterial strains by different types of probiotics and a synbiotic. In-vitro growth inhibition of *Escherichia (E.) coli EPEC, Shigella (Sh.) sonnei, Salmonella (S.) typhimurium, Klebsiella (K.) pneumoniae* and *Clostridioides (C.) difficile* were determined. Investigated products were a yeast mono strain probiotic containing *Saccharomyces (Sac.) boulardii*, bacterial mono strain probiotics containing either *Lactobacillus (L.) rhamnosus GG* or *L. reuteri DSM 17938*, a multi strain probiotic containing three *L. rhamnosus strains (E/N, Oxy, Pen)*, and a multi strain synbiotic containing nine different probiotic bacterial strains and the prebiotic fructooligosaccharides (FOS). Inhibition of pathogens was moderate by *Sac. boulardii* and *L. rhamnosus GG*, medium by *L. reuteri DSM 17938* and the *L. rhamnosus E/N, Oxy, Pen* mixture and strong by the multi strain synbiotic. Head-to-head in-vitro pathogen growth inhibition experiments can be used to differentiate products from different categories containing probiotic microorganisms and can support the selection process of products for further clinical evaluation.

## 1. Introduction

The invention of antibiotic therapy some 100 years ago was a major step forward in medical practice, allowing medical practitioners to manage otherwise deadly infections caused by pathogenic bacteria. However, in the recent past, the rapid increase of bacterial antibiotic resistance has become a pressing problem of global healthcare [1]. More sensible and less widespread use of antibiotics is necessary to counteract the increasing loss of their efficacy. While it is clinically challenging to implement, antibiotics should only be used where their usage is appropriate, and best only in confirmed cases of bacterial infections. In addition to a careful usage of antibiotics, probiotics or synbiotics should be considered as prophylactic measures, at least for patients at risk of certain bacterial infections (e.g., the elderly admitted to an intensive care unit), as complementary therapy during and after antibiotic therapy, or even as standalone therapy under certain specific circumstances for bacterial infections.

Every orally taken antibiotic is leading to alterations of the gut microbiota, in some patients with symptoms (e.g., diarrhea), in others without symptoms [2]. While antibiotic-associated diarrhea (AAD) is worrying, the disturbance of the gut microbiota by antibiotics can have more severe consequences. Among these severe side effects is, for example, pseudomembranous colitis [3], which is caused by a pathogen overgrowth of the gut, in this case by *C. difficile*. Pathogenic bacteria are omnipresent in the human gut. However, as long as the gut microbiota is well balanced and diverse, no pathogen overgrowth will take place [4]. Disturbance of the equilibrium of the gut microbiota can result in loss of this pathogenic overgrowth control. Such a can loss then lead to a strong proliferation of pathogens in the gut and finally result in disease manifestation. Supplementation with certain probiotics or synbiotics (mixtures of probiotic bacteria with a prebiotic component) during and after antibiotic therapy has been demonstrated to reduce occurrence of AAD [5].

Today, a huge variety of products containing probiotic microorganisms are available. These products can be categorized according to their composition: (i) yeast and bacterial products, (ii) mono strain and multi strain products or (iii) probiotic and synbiotic products. In the present study, the inhibitions of five bacterial pathogens, *E. coli EPEC, Sh. sonnei, S. typhimurium, K. pneumoniae* and *C. difficile* by representatives of different categories of products containing probiotic microorganisms are studied. The evaluated probiotics are the clinically well-established mono strain probiotics containing either *Sac. boulardii* [6], *L. rhamnosus GG* [7] or *L. reuteri DSM 17938* [8], and two newer products, one being a multi strain probiotic containing a mixture of three different *L. rhamnosus* strains *(E/N, Oxy, Pen)*, the other being a complex multi strain synbiotic containing six *lactobacilli* (*L. acidophilus, L. casei, L. paracasei, L. plantarum, L. rhamnosus GG, L. salivarius*), three *bifidobacteria* strains (*Bifidobacterium (B.) bifidum, B. longum, B. lactis*) and the prebiotic FOS.

Most published in-vitro growth inhibition studies focused on the characterization of only one pathogenic bacterium and one or a limited number of products containing probiotic microorganisms. The present study evaluated the antagonistic activity of a range of products in head-to-head in-vitro growth inhibition experiments with a number of different bacterial pathogens, which can support the selection of products for future, more in-depth investigations.

## 2. Materials and Methods 

### 2.1. Probiotics and Synbiotic

The yeast *Sac. boulardii* probiotic Enterol^®^ (Biocodex, Gentilly, France) contains in each capsule 4.5 × 10^9^ colony forming units (CFU) of the *Sac. boulardii* strain CNCM I-745. Dicoflor^®^ (Bayer Sp. z o.o., Warszawa, Poland) contains 6 × 10^9^ CFU of *L. rhamnosus GG* ATCC^©^ 53103 per capsule. BioGaia^®^, (InfectoPharm Arzneimittel und Consilium GmbH, Heppenheim, Germany) contains 10^8^ CFU *L. reuteri DSM 17938* per 5 drops. Lakcid^®^ (Biomed-Lublin S.A., Poland) contains a total of 2 × 10^9^ CFUs as mixture of the *L. rhamnosus* strains *E/N* (40%), *Oxy* (20%) and *Pen* (40%), [9]. The complex multi strain synbiotic Multilac^®^ Baby (Vivatrex GmbH, Aachen, Germany) is a freeze-dried powder. Each sachet contains a total of 10^9^ CFUs as a mixture of equal CFU amounts of *L. acidophilus LA-14; L. casei R0215; L. paracasei Lpc-3; L. plantarum Lp-115; L. rhamnosus GG, L. salivarius Ls-33, B. lactis Bl-04, B. bifidum R0071, B. longum R0175* and 1.43 g of the prebiotic ingredient FOS.

### 2.2. In-Vitro Growth Inhibition of E. coli EPEC, Sh. sonnei, S. typhimurium and K. pneumoniae

For the in-vitro pathogen inhibition experiments with *E. coli EPEC* (clinical isolate, collection number 3410/19 *E. coli* 025), *Sh. sonnei* ATCC^©^ 9290^TM^ (ATCC, Manassas, VA, USA), *S. enterica subsp. enterica serovar typhimurium* ATCC^©^ 14280^TM^ (ATCC, Manassas, VA, USA) and *K. pneumoniae subsp. pneumoniae* ATCC^©^ 700603^TM^ (ATCC, Manassas, VA, USA) the respective pathogen was inoculated on Columbia agar with 5% sheep blood (CM0331, Fisher Scientific GmbH, Schwerte, Germany) and incubated at 37 °C under aerobic conditions for 24 h [10]. Suspensions (100 μL) of the five tested products (mono strain probiotics *Sac. boulardii*, *L. rhamnosus GG*, *L. reuteri DSM 17938*, the multi strain *L. rhamnosus E/N, Oxy, Pen* and the complex multi strain synbiotic) each containing 10^6^ CFU were inoculated on MRS agar (CM0361, Fisher Scientific GmbH, Schwerte, Germany) and incubated for 48 h in the presence of 5% CO_2_ [11]. After the incubation, 10 mm diameter bars were cut out and transferred to a Mueller–Hinton agar (CM0337, Fisher Scientific GmbH, Schwerte, Germany) previously inoculated with the respective pathogen strain with a density of 2 on the McFarland scale. The tested cultures were stored at 4 °C for 4 h, followed by incubation at 37 °C for 24 h under aerobic conditions. 

### 2.3. In-Vitro Growth Inhibition of C. difficile

For the in-vitro pathogen inhibition studies with *C. difficile* ATCC^©^ 9689^TM^ (ATCC, Manassas, VA, USA), the pathogen was cultivated under anaerobic conditions at 35–37 °C for 24–48 h on Schaedler agar (CM0437, Fisher Scientific GmbH, Schwerte, Germany) [12]. Suspensions each containing 10^6^ CFU of the five evaluated products were inoculated on MRS agar and incubated for 48 h in the presence of 5% CO_2_. 10 mm diameter bars were transferred to a Mueller–Hinton agar with 5% horse blood and 20 mg/L NAD (PP0972, E&O Laboratories Ltd, Bonnybridge, UK) and incubated under anaerobic conditions for 24 h.

### 2.4. FOS Control and Measurement of Growth Inhibition

For testing a potential pathogen growth inhibitory effect of FOS, 100 μL of a solution containing 14.3 mg/mL FOS (F8052, Sigma Aldrich, St. Louis, Missouri, USA) was applied to a 10 mm filter disk that was then administered to respective pathogen testing plates. The multi strain synbiotic was tested on the same plates as a positive control.

At the end of the incubation, measurements of inhibition zones around the tested colonies were taken from the outer edge of the colonies to the outer edge of the clear zones. Each test was performed in triplicate and the arithmetic means of the radii measuring from the edges of the colonies to the edges of the clear zones were calculated as well as the standard deviations SD (Excel, Microsoft, Redmont, WA, USA). Independent T-test statistical analyses of datasets were conducted with GraphPad Prism software version 8.2 (GraphPad Software, San Diego, CA, USA), Datasets were considered as significantly different when a *p*-value < 0.01 was achieved.

## 3. Results

### In-Vitro Pathogen Growth Inhibition

**Figure 1:**
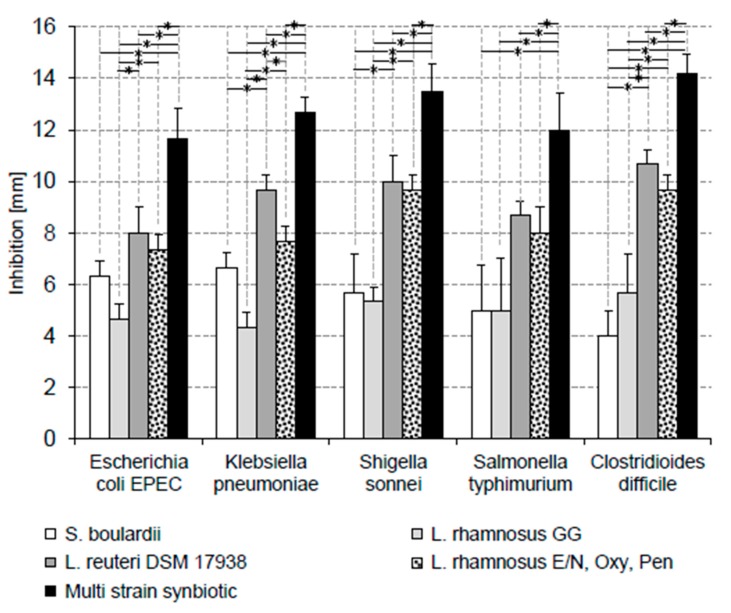
In-vitro growth inhibition of pathogens by different probiotics and a multi strain synbiotic. The *L. rhamnosus E/N, Oxy, Pen* mixture contains the three different probiotics in a CFU ratio of 40/20/40. The multi strain synbiotic contains a mixture of equal CFU amounts of *L. acidophilus LA-14; L. casei R0215; L. paracasei Lpc-3; L. plantarum Lp-115; L. rhamnosus GG, L. salivarius Ls-33, B. lactis Bl-04, B. bifidum R0071, B. longum R0175* and the prebiotic fructooligosaccharides (FOS). Significant differences (*p*-values < 0.01) between the inhibitions by different products are indicated by horizontal lines marked with an asterisk (*).

In-vitro growth of all five tested pathogenic bacteria were inhibited by all evaluated products. However, the extent of in-vitro growth inhibitions by the products varied significantly for all tested pathogens (Figure 1).

The inhibitory patterns found for *E. coli EPEC* and *K. pneumoniae* were similar. The antagonistic effects of the tested products ranked from weak to strong inhibition as follows: *L. rhamnosus GG* < *Sac. boulardii* < *L. reuteri DSM 17938* = multi strain probiotic *L. rhamnosus E/N, Oxy, Pen* < complex multi strain synbiotic. 

For the inhibition of *Sh. sonnei* and *S. typhimurium* an inhibition ranking from weak to strong of *Sac. boulardii* = *L. rhamnosus GG* < *L. reuteri DSM 17938* = multi strain probiotic *L. rhamnosus E/N, Oxy, Pen* < complex multi strain synbiotic was found. While there was a significant difference (*p*-values < 0.01) between the inhibitions of *Sh. sonnei* by *Sac. boulardii* or *L. rhamnosus GG* and the *L. reuteri DSM 17938* probiotic or the multi strain probiotic *L. rhamnosus E/N, Oxy, Pen*, the difference of the inhibitions by these two groups of probiotics of *S. typhimurium* was not significant (*p*-values > 0.01).

The inhibition of the gram-positive *C. difficile* by the tested products resembled that of *Sh. sonnei* and *S. typhimurium*. The weakest inhibitions were found for *Sac. boulardii* and *L. rhamnosus GG*. Inhibitions by *L. reuteri DSM 17938* and the multi strain probiotic *L. rhamnosus E/N, Oxy, Pen* were intermediate and the best inhibitory effect was found for the complex multi strain synbiotic.

**Figure 2:**
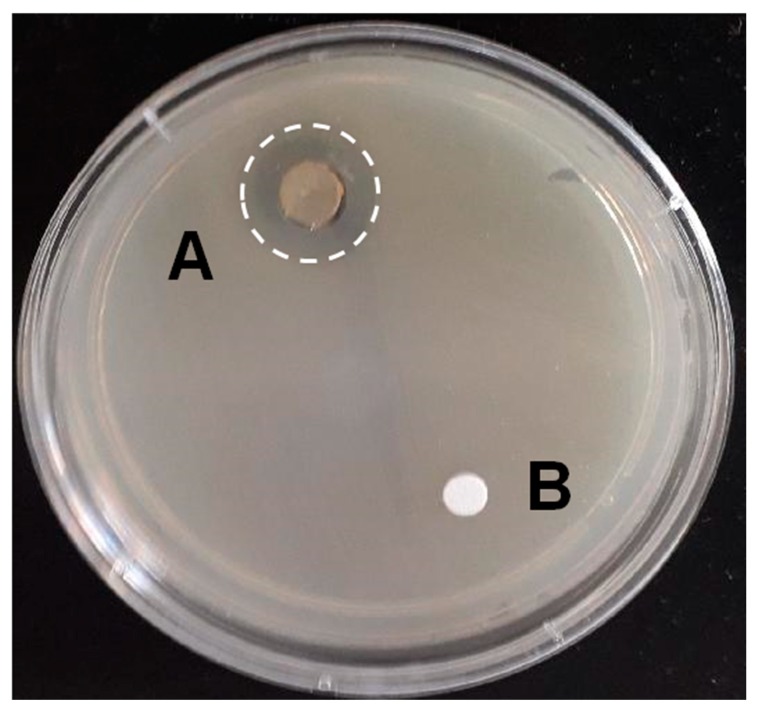
Example of the effects of the multi strain synbiotic (**A**) and FOS (**B**) on the in-vitro growth of *Enteropathogenic E. coli EPEC*.

As shown in Figure 2, FOS alone had no inhibitory effect on *E. coli EPEC*. FOS also had no inhibitory effect on the growth of the other four tested pathogens (data not shown).

## 4. Discussion

A large number of studies have been published which are characterizing the in-vitro pathogen growth effects of individual probiotic microorganisms. There are also studies which compare the growth inhibitory effects of individual strains in head-to-head experiments. Less data are available for the in-vitro growth inhibitory effect of individual multi-strain products. To our knowledge there is no study published which compares the in-vitro pathogen growth inhibitory effects of probiotic products from different categories (yeast, bacteria, mono-strain, multi-strain, probiotic, synbiotic) head-to-head. The present study compares the in-vitro pathogen growth inhibitory properties of products containing probiotic microorganisms commonly used by physicians in Poland. The in-vitro pathogen growth inhibition experiments of the present study are not aiming to identify the underlying mechanisms of growth inhibition or to identify the causes of potential synergistic effects of probiotic bacteria in the multi-strain products, but to focus primarily on establishing a ranking of efficacy in this one particular experimental set-up. Inhibition of pathogen growth is only one potential effect exhibited by probiotic bacteria in the gut and other activities (e.g., inhibition of the adherence of pathogens to the gut mucosa, stimulation of the host immune system) are also potential contributors to their efficacy. However, in-vitro pathogen growth inhibition testing is a helpful measure that can be used to support the selection of products for further investigation, e.g., in clinical trials.

For the in-vitro growth inhibition testing, five bacterial pathogens were selected which physicians encounter in their day-to-day practice. All five selected bacterial pathogens represent major health care concerns. As outlined in the following, infections with these pathogens are not always requiring antibiotic therapy. In addition, the increasing rate of developing antibiotic resistance in these bacteria has stimulated the interest in probiotics as prophylactic, alternative or adjuvant therapy for infections caused by them.

*Enteropathogenic E. coli EPEC* is a major cause of infant diarrhea [13]. As long as there is no evidence for a systemic infection, antibiotic therapy is rarely indicated and should be deferred until culture results are available. Due to this, and the emerging resistance of *E.coli* against antibiotics [14], probiotics are considered as additional options to manage *E. coli* infections [15]. In-vitro pathogen growth inhibition experiments found no clear *E. coli* growth antagonism by the yeast *Sac. boulardii* [16]. In contrast, in-vitro growth inhibitions of *E. coli* have been described for a number of mono strain bacterial probiotics, among them *L. rhamnosus GG* [17], *L. reuteri DSM 17938* [15] and multi strain probiotics [15,18,19].

*Shigella* infections are a major public health problem in areas of poor sanitation with especially high incidence, morbidity and mortality in children [20]. Shigellosis is spread by fecal-oral transmission and ingestion of a small number of *Shigella* bacteria can already cause clinical disease. Most patients recover from Shigellosis without antibiotic treatment within 5−7 days. Various antimicrobial agents are effective in the treatment of severe cases of Shigellosis, however, a globally emerging antibiotic resistance is observed [21]. Consequently, probiotics are considered as an alternative approach to manage Shigellosis. *Sac. boulardii* has been shown to interfere with *Shigella* pathogenesis [22], however, in-vitro growth inhibition of *Sh. sonnei* by *Sac. boulardii* has not been published. Inhibition of in-vitro growth of *Sh. sonnei* has been demonstrated for *L. rhamnosus GG* [17], for *L. reuteri* [23] and a number of other *lactobacilli* [24]. For the multi strain products investigated in this study, results from in-vitro growth inhibition of *Sh. sonnei* have not been demonstrated.

Most food-borne bacterial gastroenteritis is caused by *S. typhimurium*. Antibiotic therapy is not advised for most of the patients with *S. typhimurium* infection, as the infection is often self-limiting and antibiotic resistance of the bacterium is on the rise [25]. Therefore, especially for severe cases or patients with persistent infections [26], non-antibiotic treatment alternatives are needed [27]. There is a large amount of studies, including human clinical trials, indicating that *Sac. boulardii* has potential in the prevention and treatment of infections with *S. typhimurium* [28]. However, no study has been published comparing the in-vitro growth inhibition of *S. typhimurium* by *Sac. boulardii* with that of bacterial probiotics. In-vitro growth inhibition of *S. typhimurium* has been shown for a number of individual *lactobacilli* strains, [17,29,30]. The method has also been used for the search for new *lactobacilli* strains with growth inhibitory potential against *S. typhimurium* [19,31]. Our group has recently published that *S. typhimurium* was more strongly inhibited by a multi strain synbiotic (different from that investigated in the present study) when compared with the in-vitro growth inhibition caused by its individual bacterial strains [30].

*K. pneumoniae* is responsible for an alarming increase in hospital infections, especially in intensive care units. More and more antibiotics have lost their efficacy against *K. pneumoniae*, and strains of *K. pneumoniae* have emerged that are resistant against most of the presently available antibiotics [32,33]. Consequently, interest in probiotics/synbiotics as alternative options to manage infections with *K. pneumoniae* has emerged. Few studies have investigated the effect of probiotics on the in-vitro growth of *K. pneumoniae* [34,35,36], however, all with promising results.

For an initial *C. difficile* infection (CDI), the exposure to antibiotics is the most important risk factor [37]. In this context, it is important to note that certain antibiotics (e.g., clindamycin, quinolones, cephalosporins) are associated with a higher risk of causing CDI. The pathology of CDI is characterized by a disruption of the gut microbiota, resulting in an overgrowth by *C. difficile*, production of toxins and disease development. Characteristic symptoms are diarrhea and abdominal pain. In severe cases, the CDI can result in a life-threatening pseudomembranous colitis. Recurrence of CDI is a not uncommon observation. First step in the treatment of CDI is discontinuing the therapy with the inciting antibiotic as soon as possible. Depending on severity of the CDI, three antibiotics can be considered for therapy: metronidazole, vancomycin and fidaxomicin [38]. In addition, supporting the diversity of the gut microbiota is an important therapeutic objective in the management of CDI [39].

In-vitro growth inhibition of *C. difficile* by a number of mono strain probiotics, among them *L. rhamnosus*, and *bifidobacteria* has been described [40,41]. To our knowledge, the present study is the first which compares head-to-head the in vitro-growth inhibition of *C. difficile* by representatives from different categories of products containing probiotics.

The products evaluated in the present study can be categorized by the following criteria: (i) yeast/bacteria, (ii) mono strain/multi strain and (iii) probiotic/synbiotic (containing probiotic and prebiotic components). While the in-vitro pathogen growth inhibitory effects vary among the tested products, the inhibitory effects seem not to be pathogen-dependent. Weakest pathogen inhibition is observed for *Sac. boulardii* and *L. rhamnosus GG*, intermediate for *L. reuteri DSM 17938* and the *L. rhamnosus E/N, Oxy, Pen* mixture and strongest inhibition is found for the multi strain synbiotic. This finding is in line with the hypothesis that multi strain probiotics exhibit superior growth inhibitory effects towards pathogenic bacteria because they are combining a broader range of independent antibacterial activities, some of which might even act synergistically [42,43]. However, it has to be mentioned that in-vitro characterization of the potential synergistic effects in multi strain probiotics, especially those containing a larger number of different strains, can hardly be demonstrated due to the fact that thousands, if not millions of potential combinations would have to be investigated. Synbiotics might have an additional advantage over pure probiotics, as their prebiotic component provides a source of energy, potentially supporting the proliferation of their probiotic components. Based on our experiments, we can exclude that FOS itself has an inhibitory effect on one of the tested pathogenic bacteria.

An obvious limitation of the present study is that it is using only one experimental set-up to establish an in-vitro efficacy ranking among the evaluated products. No efforts have been undertaken to investigate the underlying mechanisms of the observed pathogen inhibitions or the underlying mechanisms of potential synergies in the multi strain products. Dedicated human clinical studies will be necessary to evaluate if these in-vitro findings will translate into clinical benefits for patients.

## 5. Conclusions

In-vitro growth inhibition of a variety of pathogens is helpful to differentiate products and product categories containing probiotic microorganisms. Based on the results of the present study, multi strain probiotics should be preferred in case a strong in-vitro growth inhibition of a broad range of pathogens is desired. Our study results can support the product selection for future clinical investigations.
